# LPCAT1 and MRPL9 Promote Hepatocellular Carcinoma Progression via AKT Phosphorylation and Define a Mitochondrial Prognostic Model

**DOI:** 10.3390/cancers18071144

**Published:** 2026-04-02

**Authors:** Hui Yang, Ziqi Zhu, Hanwen Zhu, Nanjing Li, Sinian Zheng, Ling Hu, Zhenru Wu, Yujun Shi

**Affiliations:** 1 Department of Pathology & Institute of Clinical Pathology, Institute of Transplantation, Key Laboratory of Transplant Engineering and Immunology, NHC, West China Hospital, Sichuan University, Chengdu 610041, China; 2 West China School of Medicine, Sichuan University, Chengdu 610041, China; 3 Division of Radiotherapy, Cancer Center, West China Hospital, Sichuan University, Chengdu 610041, China

**Keywords:** hepatocellular carcinoma, mitochondrial-related genes, immunotherapy, prognosis

## Abstract

**Background/Objectives:** Mitochondrial dysfunction plays a critical role in hepatocellular carcinoma (HCC) progression. This study aimed to identify mitochondrial-related prognostic genes and elucidate their functional mechanisms in HCC. **Methods:** Mitochondrial-related prognostic genes were screened from the TCGA and ICGC-LIHC cohorts. A prognostic risk model was constructed and validated in three external cohorts. Immunotherapy sensitivity between high- and low-risk groups was assessed using somatic mutation and immune infiltration analyses. Underlying molecular mechanisms were further validated through in vitro experiments in HCC cell lines. **Results:** Transcriptome analysis identified 15 mitochondrial-related prognostic genes, from which 10 candidates were refined using LASSO regression. Among them, LPCAT1 and MRPL9 were significantly associated with overall survival. The 2-MRG model (comprising LPCAT1 and MRPL9) demonstrated superior prognostic accuracy and better survival stratification across both internal and external cohorts. Functional enrichment analysis revealed significant metabolic dysfunction in the high-risk group. The high-risk group also exhibited a significantly higher tumor mutation burden and an enhanced predicted responsiveness to immunotherapy. In vitro, knockdown of MRPL9 or LPCAT1 inhibited the proliferation and migration of HCC cells by inducing G1/S arrest and G2/M arrest, respectively. Moreover, they also suppressed the AKT phosphorylation. **Conclusions:** We developed a robust 2-MRG prognostic model based on LPCAT1 and MRPL9 that effectively predicts HCC outcomes and supports individualized therapeutic decision-making. Both genes promote HCC progression by modulating the AKT phosphorylation and cell-cycle checkpoints, highlighting their potential as therapeutic targets.

## Simple Summary

Hepatocellular carcinoma (HCC) lacks reliable biomarkers for prognosis and actionable targets for therapies, prompting the need to identify molecules that can improve risk stratification and guide treatment decisions. We found two genes related to the mitochondria, LPCAT1 and MRPL9, developed and validated a model for prognosis, and demonstrated that both genes promote HCC progression by controlling AKT phosphorylation and cell-cycle checkpoints. These findings provide an applicable tool for prognosis and highlight LPCAT1 and MRPL9 as potential targets for therapies. We offered new avenues for improving patient management and individualized therapy in HCC.

## 1. Introduction

Hepatocellular carcinoma (HCC), accounting for approximately 90% of all primary liver cancer [1], is a major global health burden and ranks as the third leading cause of cancer-related mortality worldwide [2]. Systemic treatment for HCC has evolved substantially with the development of targeted therapies, such as multi-tyrosine kinase inhibitors like sorafenib and lenvatinib [3,4], as well as immune checkpoint inhibitors including atezolizumab plus bevacizumab [5,6] and Tremelimumab plus Durvalumab [7,8]. These agents enhance anti-tumor immune responses by blocking immunosuppressive pathways that hinder effector lymphocyte activation. However, durable clinical benefits are achieved in only a limited subset of patients, largely due to late-stage diagnosis, high recurrence rates, and substantial tumor heterogeneity [9,10,11,12]. Therefore, there is an urgent need for robust and reliable biomarkers to predict disease progression, facilitate precise risk stratification, and guide personalized therapeutic decision-making in HCC. Although biomarkers such as serum alpha-fetoprotein [13,14] and staging systems such as the Barcelona Clinic Liver Cancer (BCLC) and TNM [10,15] are widely used, they are insufficient to reflect the molecular complexity and biological diversity of HCC.

Mitochondria, beyond their classical role in energy production, have been increasingly recognized as central regulators of cellular metabolism, redox homeostasis, apoptosis, and immune evasion [16]. Recent studies have shown that mitochondrial dysfunction in tumors can lead to metabolic reprogramming, elevated reactive oxygen species production, and remodeling of the tumor immune microenvironment, thereby inducing aggressive tumor phenotypes and facilitating immune escape [17,18,19]. These findings underscore the critical role of mitochondrial dysregulation in cancer progression. However, the diagnostic, prognostic, and therapeutic relevance of mitochondrial-related genes (MRGs) in HCC remains unclear. A deeper understanding of MRGs may provide novel biomarkers and support the development of precision therapeutic strategies for HCC management. Mitochondrial ribosomal proteins (MRPs) play a pivotal role in maintaining the structural and functional integrity of the mitochondrial ribosome. The MRP gene family comprises MRPL genes, which encode proteins of the large ribosomal subunit, and MRPS genes, which encode proteins of the small ribosomal subunit [20]. MRPL9 encodes a component of the mitochondrial large ribosomal subunit [21], and previous studies have demonstrated its involvement in mitochondrial translation, apoptosis, and cellular energy metabolism [22,23]. Lysophosphatidylcholine acyltransferase 1 (LPCAT1) catalyzes the conversion of lysophosphatidylcholine to phosphatidylcholine [24], a key process in membrane phospholipid remodeling through the Lands cycle. Increasing evidence suggests that aberrant LPCAT1 expression contributes to tumor cell proliferation and metastasis [25,26].

In this study, bioinformatic analyses of multi-cohort RNA-seq datasets and corresponding clinical information from HCC patients identified two key mitochondrial-related genes, LPCAT1 and MRPL9, as potential prognostic biomarkers. Based on these genes, we constructed a robust risk score model and assessed its ability to predict immunotherapy responsiveness. The functional roles of LPCAT1 and MRPL9 in HCC progression were further validated in vitro.

## 2. Materials and Methods

### 2.1. Data Collection and Processing

RNA-seq, clinical, and somatic mutation data for liver hepatocellular carcinoma (LIHC) were retrieved from The Cancer Genome Atlas (TCGA) database and served as the derivation cohort. After matching the RNA-seq and clinical data, a total of 351 hepatocellular carcinoma (HCC) tissue samples and 49 adjacent non-tumor tissue samples were included. Four HCC samples with a survival time of less than one day were excluded from further analysis. The somatic mutation dataset included 369 HCC samples. RNA-seq and clinical data from the International Cancer Genome Consortium (ICGC) database for LIHC were used as an internal validation cohort, comprising 212 HCC samples and 177 adjacent samples. External validation was performed using RNA-seq and clinical data from the Gene Expression Omnibus (GEO) database, including the GSE14250 dataset (225 HCC and 220 adjacent samples) and the GSE124751 dataset (206 HCC samples). Additionally, RNA-seq and clinical data from the OEP00000321 dataset, comprising 158 HCC samples and 158 adjacent samples, were obtained from the National Omics Data Encyclopedia (NODE) database as an additional external validation cohort. GSE25097 data, including RNA-seq and clinical data from the dataset (6 healthy, 40 cirrhotic, 243 adjacent, and 268 HCC samples), were collected from the GEO database. LIHC protein data were downloaded from the Clinical Proteomic Tumor Analysis Consortium (CPTAC) database. To ensure comparability across cohorts, raw expression data were log2-transformed when necessary and normalized prior to downstream analysis. For microarray datasets, normalization between arrays was performed using the “normalizeBetweenArrays” function in the R package limma (R version 4.5.0). For RNA-seq datasets, expression values were transformed to transcripts per million (TPM) format and log2 (TPM + 1) scaled to reduce technical variability.

### 2.2. Screening of Mitochondrial-Related Prognostic Biomarkers

To identify mitochondrial-related prognostic biomarkers in HCC, univariate Cox regression analysis was performed using transcriptomic data from the TCGA-LIHC and ICGC cohorts to screen for genes significantly associated with overall survival (OS) (*p* < 0.05). A list of 547 mitochondrial-related genes (MRGs) was collected from the GeneCards database and the previously published literature. A Venn diagram was employed to identify overlapping genes between OS-associated genes and the MRG list, thereby defining mitochondrial-related prognostic candidates. To avoid overfitting, LASSO-Cox regression was performed on the TCGA-LIHC dataset using the R package glmnet, thereby identifying the most robust prognostic MRG features. A LASSO-Cox regression model was applied, with gene expression levels as independent variables and patients’ survival time and status as outcome variables. The optimal penalty parameter (λ) was determined through 10-fold cross-validation using the glmnet function across a predefined sequence of λ values. The λ value corresponding to the minimum cross-validated partial likelihood deviance (lambda.min) was selected. The model was then refitted using this optimal λ, and genes with non-zero coefficients were retained as prognostic feature genes.

### 2.3. Development and Validation of MRG-Related Prognostic Model for Patients with HCC

A multivariate Cox proportional risk model was constructed based on the Akaike Information Criterion for forward and backward stepwise selection. Two prognostic MRGs (LPCAT1 and MRPL9; *p* < 0.05, multivariate Cox regression analysis) were identified as significant prognostic MRGs, and risk score models were developed based on these MRGs. The prognostic risk score was calculated using the following formula:

$$\mathrm{Risk}\;\mathrm{score}=\sum_{i}^{n}({\mathrm{Coef}}_{i}\times A_{i})$$

where Coefᵢ is the regression coefficient of gene i in the multivariate Cox model, Aᵢ represents the normalized expression level of gene i, and n is the total number of genes included in the prognostic signature. Patients were stratified into high- and low-risk groups based on the median risk score cutoff value. Kaplan–Meier survival curves were generated to compare overall survival between risk groups, and the log-rank test was used to evaluate statistical significance. The prognostic performance of the model was further assessed using time-dependent receiver operating characteristic (ROC) curves, providing a dynamic evaluation of model accuracy over time. Kaplan–Meier survival curves and time-dependent ROC curves were also performed in the internal (ICGC) and external (OEP0003221, GSE14250, and GSE124751) validation cohorts.

### 2.4. Differential Gene Expression and Functional Enrichment Analysis

Differentially expressed genes (DEGs) between groups were identified using the DESeq2 package in R. Genes with an |log2-fold change| > 0.5 and an adjusted *p*-value < 0.05 were considered statistically significant. Gene Ontology (GO) functional analysis and Kyoto Encyclopedia of Genes and Genomes (KEGG) pathway analysis were performed based on DEGs. Gene Set Enrichment Analysis (GSEA) was performed using pre-ranked gene lists, focusing on GO biological processes and Kyoto KEGG pathways. Gene sets were obtained from the Molecular Signatures Database (MSigDB).

### 2.5. Somatic Mutation Analysis

Somatic mutation profiles were analyzed using the R package “maftools” to compare the mutational landscape between the high- and low-risk groups. Differences in mutation frequency and patterns were assessed to identify risk group-specific genomic alterations.

### 2.6. Immune Cell Infiltration Analysis

The infiltration status of 22 immune cell types in TCGA-LIHC samples was evaluated using the CIBERSORT algorithm via the R package IOBR. Comparisons of immune cell infiltration between the high- and low-risk groups were performed using the Wilcoxon rank-sum test. Spearman correlation analysis was conducted to assess the relationships between the 2-MRG risk score or gene expression levels and immune cell proportions.

### 2.7. Immunohistochemistry Analysis

To confirm higher protein levels of LPCAT1 and MRPL9 in HCC tissues compared with normal tissues, we used immunohistochemical results from the HPA database.

### 2.8. Cell Culture and Transfection

The mouse hepatocellular carcinoma cell line (Hepa16) and human hepatocellular carcinoma cell line (Huh7) (Cell Bank of the Chinese Academy of Science, Shanghai, China) were cultured in Dulbecco’s modified Eagle’s medium (DMEM) (Gibco, Grand Island, NY, USA) supplemented with 10% fetal bovine serum (FBS) (Gibco) and 1% antibiotics (penicillin and streptomycin) (Gibco). Cells were maintained at 37 °C in a humidified incubator containing 5% CO_2_.

For transfection experiments, cells were seeded into 6-well plates at a density of 2 × 10^5^ cells per well and allowed to adhere overnight to reach approximately 60–70% confluence. Small interfering RNAs (siRNAs) targeting MRPL9 or LPCAT1, and the corresponding negative control siRNA, were purchased from GenePharma (Shanghai, China). The final siRNA concentration used for transfection was 20 nM. Transfection was performed using Lipofectamine RNAiMAX (Thermo Fisher Scientific, Waltham, MA, USA) according to the manufacturer’s instructions. Briefly, siRNA and Lipofectamine RNAiMAX were diluted separately in Opti-MEM reduced-serum medium (Gibco), combined, and incubated at room temperature for 10–15 min to allow complex formation before being added to the cells. After 24 h of incubation, the transfection medium was replaced with complete DMEM containing 10% FBS. Cells were harvested 24–48 h post-transfection for subsequent experiments. Knockdown efficiency was verified by quantitative real-time PCR and/or Western blot analysis.

### 2.9. EdU Assay

Cell proliferation was assessed using the EdU Cell Proliferation Kit with Alexa Fluor 488 (Beyotime, Shanghai, China) following the manufacturer’s instructions. Briefly, Hepa1-6/Huh7 cell lines were cultured with EdU (10 μm) for 24 h at 37 °C. Then, cells were fixed with 4% formalin, permeabilized with 0.5% Triton X-100, and the incorporated EdU was detected via a Click Additive Solution with Alexa Fluor 488. Flow cytometry and immunofluorescence were used to assess EdU-positive cells.

### 2.10. Cell Migration Assay

Hepa1-6/Huh7 cell lines were grown to confluence in 6-well plates, and a uniform scratch was created with a sterile pipette tip. After washing, fresh medium was added, and cell migration into the wound area was monitored by microscopy at 0 and 24 h. The wound area was measured in ImageJ (Version 2021), and the percentage of wound closure was calculated.

### 2.11. Cell Cycle Assay

Cycle Staining Kit (Beyotime) was used according to the manufacturer’s protocol. Briefly, Hepa1-6/Huh7 cell lines were harvested and fixed in 70% ethanol overnight at 4 °C, then treated with RNase A and propidium iodide (PI) at 37 °C in the dark for 30 min.

### 2.12. Real-Time Quantitative PCR (qRT-PCR)

Total RNA was extracted from hepatocellular carcinoma (HCC) cell lines using TRIzol reagent, following the manufacturer’s instructions. The quality and concentration of the RNA samples were measured using a NanoDrop 2000 spectrophotometer (Thermo Fisher Scientific). cDNA was synthesized from the extracted RNA using the HiScript II Q RT SuperMix for qPCR (+gDNA) (Vazyme, Nanjing, China). Quantitative RT-PCR was then performed using the ChamQ Universal SYBR Master Mix (Vazyme). Accordingly, qRT-PCR was performed using the ProFlex Base (Thermo Fisher Scientific) and the 7500 Real-Time PCR System (Thermo Fisher Scientific). Gene expression levels were normalized to the housekeeping gene GAPDH, with target primer sequences listed in Table S3. Relative expression levels were calculated using the 2−ΔΔCt method, with samples from the NC group serving as the reference for normalization.

### 2.13. Western Blotting

Treated cell lines were lysed in RIPA buffer (Beyotime) containing phosphatase inhibitors (Roche, Basel, Switzerland) and protease inhibitors (Roche). Proteins were separated on ExpressPlus PAGE gels (Genscript, Nanjing, China) and transferred onto nitrocellulose membranes according to the manufacturer’s instructions. Membranes were blocked with QuickBlock (Beyotime) and then incubated overnight at 4 °C with primary antibodies against LPCAT1, MRPL9, p-AKT, AKT, and GAPDH. After five washes with Tris-Buffered Saline containing Tween-20 (TBST), membranes were incubated for 1 h at room temperature with HRP-conjugated secondary antibodies (ABclonal Technology, 1:50,000 dilution, Wuhan, China). Protein detection was carried out using Super ECL Detection Reagent (Yeasen, Shanghai, China), and the immunoblots were visualized using an imaging system. The specific antibodies used are listed in Table S4.

### 2.14. Statistical Analysis

All results are presented as mean ± standard error of the mean (SEM). Statistical analyses were performed using R (version 4.5.0). For comparisons between two groups of continuous variables, either Student’s *t*-test or the Mann–Whitney U test was applied, depending on data distribution. The one-way ANOVA test was used for comparisons among multiple groups. Statistical significance was indicated as follows: * *p* < 0.05; ** *p* < 0.01; *** *p* < 0.001; **** *p* < 0.0001.

## 3. Results

### 3.1. Identification of Key Mitochondrial-Related Prognostic Biomarkers for HCC

**Figure 1:**
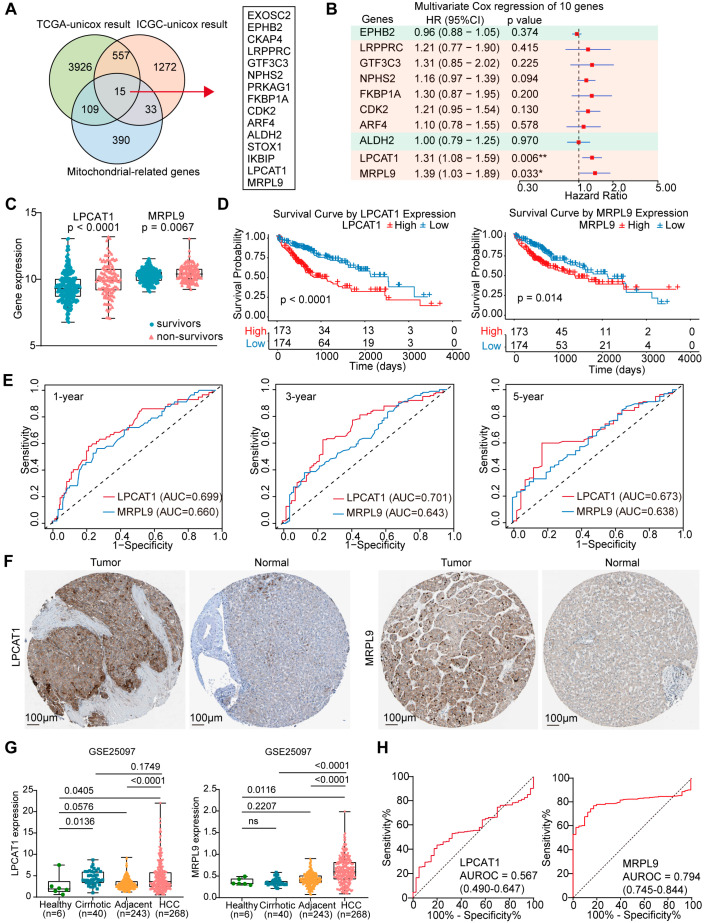
Identification of key mitochondrial-related prognostic biomarkers for HCC. (**A**) Venn diagram illustrating the 15 overlapping mitochondrial-related prognostic genes identified from univariate Cox regression analyses in the TCGA and ICGC cohorts, along with the mitochondrial-related gene dataset. (**B**) Multivariate Cox regression analysis of the ten featured genes. * *p* < 0.05; ** *p* < 0.01. (**C**) Expression levels of LPCAT1 and MRPL9 in HCC survivors versus non-survivors. (**D**) Kaplan–Meier survival curves comparing overall survival between high- and low-expression groups, stratified by the median expression cutoff of LPCAT1 and MRPL9. (**E**) ROC curves assessing the predictive performance of LPCAT1 and MRPL9 expression levels for 1-, 3-, and 5-year overall survival. (**F**) Immunohistochemical staining of LPCAT1 and MRPL9 in HCC tumor tissues and normal tissues from the HPA database. (**G**) LPCAT1 and MRPL9 expression across healthy, cirrhotic, adjacent, and HCC tissues in the GSE25097 cohort. (**H**) ROC curves of LPCAT1 and MRPL9 expression for distinguishing HCC from cirrhotic patients in the GSE25097 cohort. HR: Hazard Ratio; AUC: Area Under the Curve; AUROC: Area Under the Receiver Operating Characteristic curve.

Univariate Cox regression analysis of hepatocellular carcinoma (HCC) transcriptomic data from the TCGA and ICGC cohorts identified 4608 and 1877 genes, respectively, that were significantly associated with overall survival (Tables S1 and S2, respectively). Venn diagram analysis integrating these results with the mitochondrial-related gene set identified 15 overlapping genes as key mitochondrial-related prognostic markers (Figure 1A). We then used LASSO-Cox regression analysis to screen the 10 featured genes (Figure S1). Multivariate Cox regression analysis showed that LRPPRC, GTF3C3, NPHS2, FKBP1A, CDK2, ARF4, LPCAT1, and MRPL9 were risk factors for HCC patients, while EPHB2 and ALDH2 were protective factors (Figure 1B). Among these genes, only LPCAT1 and MRPL9 were significantly associated with HCC prognosis (Figure 1B). In the TCGA cohort, both LPCAT1 and MRPL9 expression levels were significantly elevated in non-survivors compared to survivors (Figure 1C). Patients were stratified into high- and low-expression groups based on the median expression of each gene. Kaplan–Meier survival analysis indicated that patients in the low-expression group had significantly improved overall survival compared with those in the high-expression group (Figure 1D). ROC analysis demonstrated favorable prognostic accuracy for LPCAT1 (AUC = 0.699, 0.701, and 0.673) and MRPL9 (AUC = 0.660, 0.643, and 0.638) at 1-, 3-, and 5-year, respectively (Figure 1E). Immunohistochemical analysis further confirmed the elevated protein expression of LPCAT1 and MRPL9 in HCC tissues compared to normal tissues (Figure 1F). Additionally, analysis of the GSE25097 cohort revealed that LPCAT1 expression was significantly higher in HCC tissues than in adjacent or healthy liver tissues, and was also elevated in cirrhotic samples versus healthy controls (Figure 1G). MRPL9 expression was significantly increased in HCC samples compared to healthy, cirrhotic, and adjacent tissues (Figure 1G). Notably, ROC analysis showed limited diagnostic performance for LPCAT1 in distinguishing HCC from cirrhotic patients (AUC = 0.567), whereas MRPL9 demonstrated robust discriminatory power (AUC = 0.794), underscoring its potential as a diagnostic biomarker (Figure 1H). These findings highlight the diagnostic and prognostic value of LPCAT1 and MRPL9 in HCC, suggesting their potential utility as biomarkers for early detection and outcome prediction.

### 3.2. Development and Validation of a Prognostic Risk Score Model

**Figure 2:**
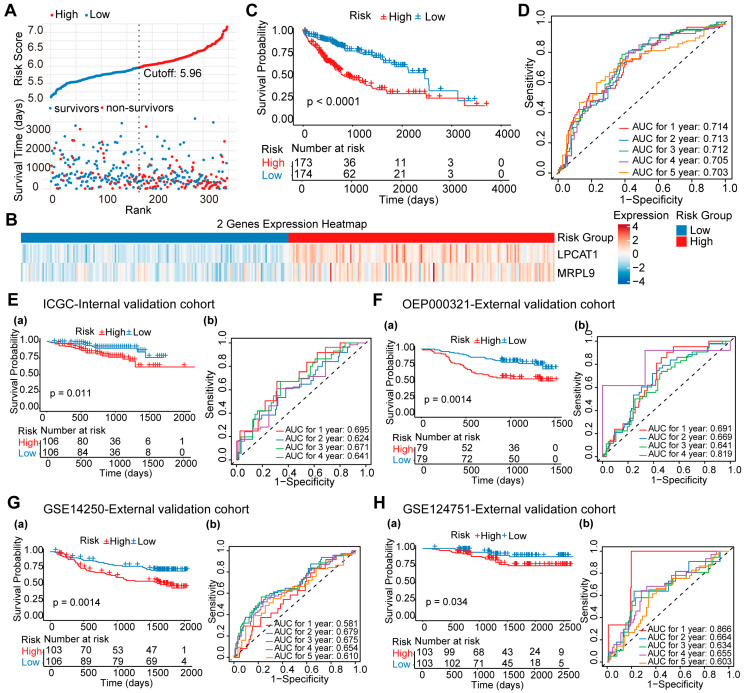
Development of two mitochondrial-related genes (MRGs) risk score models in the TCGA-LIHC cohort. (**A**) Classification of HCC patients into high-risk and low-risk groups based on the median 2-MRG (LPCAT1 and MRPL9) risk score cutoff. (**B**) Expression levels of the 2-MRG signature in the high-risk and low-risk groups. (**C**) Kaplan–Meier survival curves comparing overall survival between the high-risk and low-risk groups for the 2-MRG risk score model. (**D**) Time-dependent ROC curves assessing the predictive accuracy of the 2-MRG risk score model for 1-, 2-, 3-, 4-, and 5-year survival. Kaplan–Meier survival curves comparing overall survival between high-risk and low-risk groups based on the 2-MRG risk score model (**E**(**a**)) in the ICGC Internal validation cohort, (**F**(**a**)) in the OEP000321 external validation cohort, (**G**(**a**)) in the GSE14520 External validation cohort, and (**H**(**a**)) in the GSE76427 external validation cohort. Time-dependent ROC curves assessing the predictive accuracy of the 2-MRG risk score model for 1-, 2-, 3-, 4-, and 5-year survival (**E**(**b**)) in the ICGC Internal validation cohort, (**F**(**b**)) in the OEP000321 external validation cohort, (**G**(**b**)) in the GSE14520 External validation cohort, and (**H**(**b**)) in the GSE76427 external validation cohort. AUC: Area Under the Curve.

Based on the two significant prognostic MRGs (LPCAT1 and MRPL9), we developed a risk score model using a TCGA derivation cohort and validated it in both internal and external cohorts. The 2-MRG risk prognostic model for HCC patients was constructed via multivariate Cox regression in the TCGA cohort and calculated using the following formula: Risk score = 0.269 × LPCAT1 + 0.33 × MRPL9. Patients were divided into high- and low-risk groups based on the median risk score cutoff of 5.96 (Figure 2A). Non-survivors were mainly concentrated in the high-risk group, where both LPCAT1 and MRPL9 were significantly upregulated (Figure 2A,B). Kaplan–Meier survival analysis showed a significant improvement in overall survival in the low-risk group (Figure 2C). These findings were validated in the ICGC internal cohort as well as in the OEP000321, GSE14250, and GSE124751 external cohorts (Figure 2E–H(a)). Time-dependent ROC analysis showed reliable predictive performance at 1-, 2-, 3-, 4-, and 5-year survival (AUROC = 0.714, 0.713, 0.712, 0.705, and 0.703, respectively) (Figure 2D), with validation across all cohorts (Figure 2E–H(b)). These findings highlight that the 2-MRG risk score model, with its simplicity and enhanced cross-cohort applicability, may serve as a more practical and robust prognostic tool in HCC.

### 3.3. Functional Characteristics of Risk Groups in the 2-MRG Risk Score Model

To explore the biological processes underlying the prognostic differences between high- and low-risk groups defined by the 2-MRG risk score model, we conducted functional enrichment analyses to characterize their distinct molecular profiles. Differential expression analysis identified 741 significantly upregulated and 144 downregulated differentially expressed genes (DEGs) in the high-risk group compared with the low-risk group (|log2-fold change| > 0.5; adjusted *p*-value < 0.05) (Figure S2).

**Figure 3:**
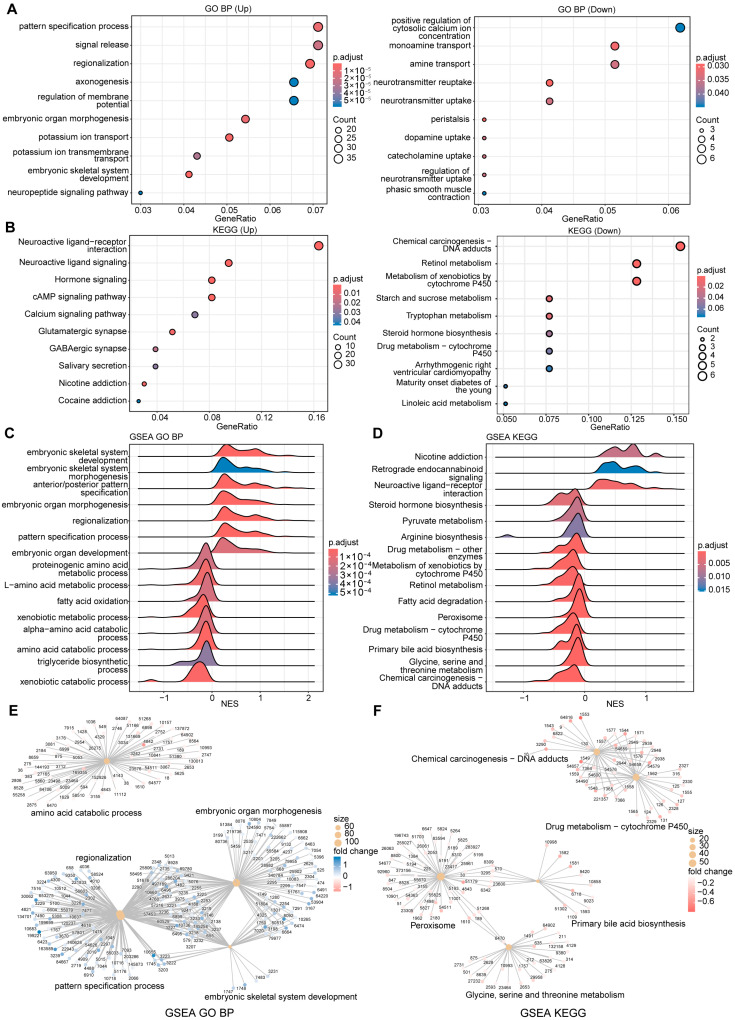
Functional analysis of risk groups in the 2-MRG risk score model. (**A**) Top 10 GO biological processes significantly up- or downregulated in the high- versus low-risk groups. (**B**) Top 10 significantly upregulated and downregulated KEGG pathways between high- and low-risk groups. (**C**,**D**) GSEA showing the top 15 significantly enriched (**C**) GO biological processes and (**D**) KEGG pathways in the high- versus low-risk groups. (**E**,**F**) Category network plots displaying the top 5 significantly enriched (**E**) GO biological processes and (**F**) KEGG pathways based on GSEA results. GO: Gene Ontology; KEGG: Kyoto Encyclopedia of Genes and Genomes; GSEA: gene set enrichment analysis.

Gene Ontology (GO) functional analysis, Kyoto Encyclopedia of Genes and Genomes (KEGG) pathway analysis based on DEGs, and pre-ranked gene set enrichment analysis (GSEA) were conducted to explore correlated pathways. The results revealed significant downregulation of liver metabolism-related pathways in the high-risk group, including those involved in catabolic processes, biosynthetic pathways, and xenobiotic metabolism (Figure 3A–D). These alterations align with the impaired hepatic function typically seen in advanced liver disease. The category network plot further highlighted the interconnections between key enriched pathways and core genes identified by GSEA, emphasizing the loss of metabolic functionality in high-risk individuals (Figure 3E,F). These findings suggest that the high-risk group identified by the 2-MRG risk score model is characterized by substantial metabolic dysfunction, underscoring its value in prognostic stratification of HCC patients.

### 3.4. The 2-MRG Risk Score Model Associated with Disease Severity

**Figure 4:**
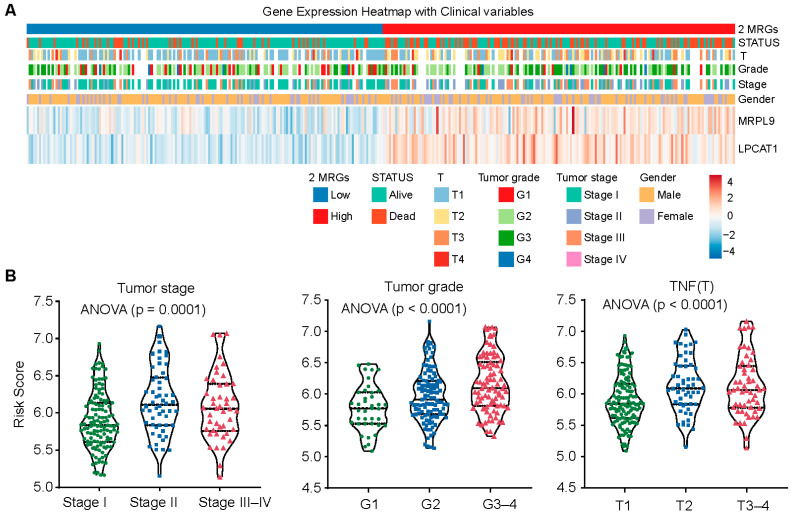
Correlation of the 2-MRG risk score model and disease severity. (**A**) Heatmap showing the distribution of the 2-MRG risk score, gene expression levels (LPCAT1 and MRPL9), and associated clinical variables in the TCGA cohort. (**B**) Violin plots illustrating the distribution of 2-MRG risk scores across tumor stages, grades, and sizes, highlighting associations between risk scores and clinical variables. The lines in the Violin indicate the median as well as the first and third quartiles.

To assess the clinical relevance of the 2-MRG risk score model, we investigated its association with disease severity. Stratification based on clinical characteristics revealed that higher 2-MRG risk scores were significantly associated with advanced tumor stage, higher histological grade, larger tumor size, and poorer clinical outcomes, with both LPCAT1 and MRPL9 highly expressed in the high-risk group (Figure 4A,B). These findings underscore a strong correlation between the 2-gene signature and disease severity.

### 3.5. Immunotherapy Sensitivity in Risk Groups

**Figure 5:**
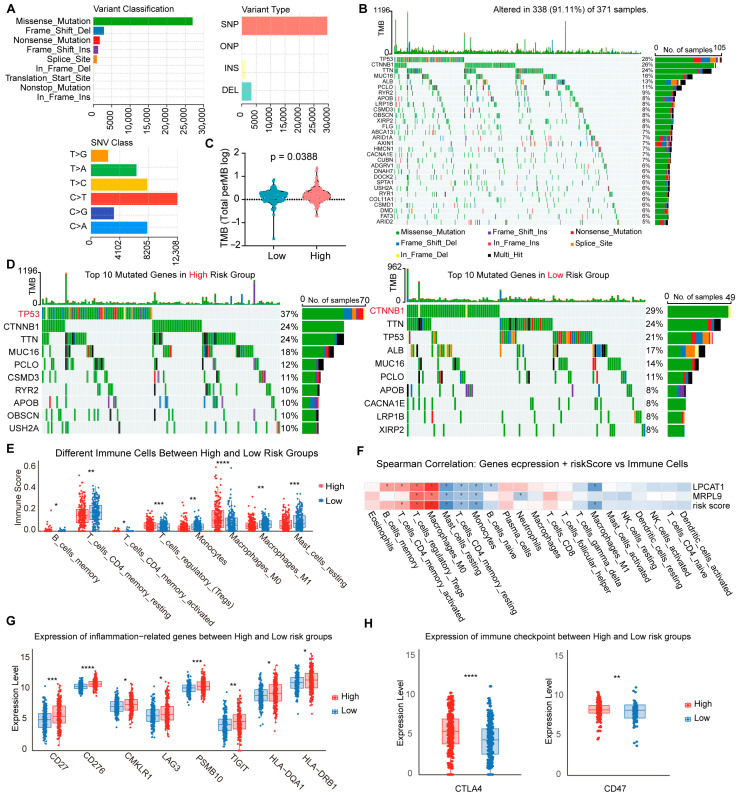
Immune response and immune checkpoint sensitivity in 2-MRG risk groups. (**A**) Somatic mutation landscape of HCC in the TCGA-LIHC cohort. (**B**) Top 30 most frequently mutated genes in HCC. (**C**) Comparison of tumor mutation burden (TMB) between high-risk and low-risk groups. (**D**) Waterfall plots illustrating the top 10 mutated genes in the high-risk and low-risk groups, respectively. (**E**) Violin plot displaying differences in immune cell infiltration between high- and low-risk groups. (**F**) Correlation analysis of the 2-MRG risk score and expression levels of LPCAT1 and MRPL9 with immune cell infiltration levels. (**G**) Expression levels of inflammation-related genes in high- versus low-risk groups. (**H**) Expression levels of immune checkpoint genes in high- versus low-risk groups. * *p* < 0.05; ** *p* < 0.01; *** *p* < 0.001; **** *p* < 0.0001.

To assess potential differences in immunotherapy response, we evaluated somatic mutation profiles, immune cell infiltration, and the expression of inflammation- and immune checkpoint-related genes across the high- and low-risk groups defined by the 2-MRG risk score model. Somatic mutation analysis in HCC patients revealed that missense mutations were the most common variant classification, and single-nucleotide polymorphisms (SNPs), particularly C > T transitions, were the predominant variant type (Figure 5A). The top 10 frequently mutated genes included TP53, CTNNB1, TTN, MUC16, ALB, PCLO, RYR2, APOB, LRP1B, and CSMD3 (Figure 5B). Tumor mutation burden (TMB) analysis revealed a significantly higher TMB in the high-risk group compared to the low-risk group (Figure 5C). Notably, CTNNB1 mutations were more prevalent in the low-risk group, while TP53 mutations occurred more frequently in the high-risk group (Figure 5D), suggesting distinct mutational landscapes that may influence immunotherapy responses.

Subsequent immune infiltration analysis revealed differences in immune cell infiltration levels between the high- and low-risk groups (Figure 5E). Pearson correlation analysis indicated that both the prognostic genes and the risk score were significantly associated with infiltrating immune cells (Figure 5F). Moreover, the expression of inflammation-related genes was significantly upregulated in the high-risk group (Figure 5G). In addition, several immune checkpoint genes, including CTLA4 and CD47, were significantly upregulated in the high-risk group (Figure 5H). These findings indicate that the high-risk group displays unique immune-related characteristics, potentially enhancing their responsiveness to immunotherapy. Accordingly, the 2-MRG risk score model may serve not only as a prognostic indicator but also as a valuable tool for informing immunotherapeutic decision-making in HCC.

### 3.6. LPCAT1 and MRPL9 Knockdown Represses the Proliferation and Migration of HCC Cells

**Figure 6:**
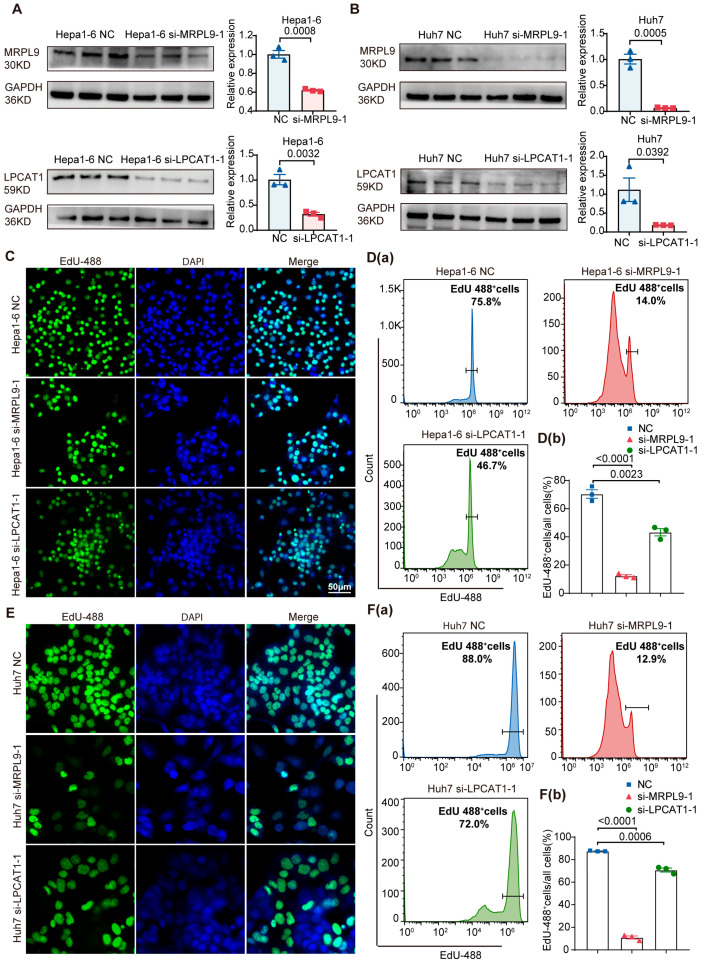
Effects of LPCAT1 and MRPL9 on the proliferation of hepatocellular carcinoma cells. (**A**) Western blot and qRT-PCR analyses of MRPL9 and LPCAT1 expression in Hepa1-6 NC, Hepa1-6 siMRPL9-1, and Hepa1-6 siLPCAT1-1 cells. (**B**) Western blot and qRT-PCR analyses of MRPL9 and LPCAT1 expression in Huh7 NC, Huh7 siMRPL9-1, and Huh7 siLPCAT1-1 cells. (**C**) Immunofluorescence staining of EdU incorporation (green) with DAPI nuclear staining (blue) in Hepa1-6 NC, Hepa1-6 siMRPL9-1, and Hepa1-6 siLPCAT1-1 cells. Scale bar: 50 µm. (**D**(**a**,**b**)) Cytometric analysis of the EdU-positive cells in the Hepa1-6 NC, Hepa1-6 siMRPL9-1, and Hepa1-6 siLPCAT1-1 groups. (**E**) Immunofluorescence staining of EdU incorporation (green) with DAPI staining (blue) in Huh7 NC, Huh7 siMRPL9-1, and Huh7 siLPCAT1-1 cells. Scale bar: 50 µm. (**F**(**a**,**b**)) Cytometric analysis of the EdU-positive cells in Huh7 NC, Huh7 siMRPL9-1, and Huh7-1 siLPCAT1-1 groups. Mean ± SEM, *n* = 3/group. Original western blots are presented in File S1.

**Figure 7:**
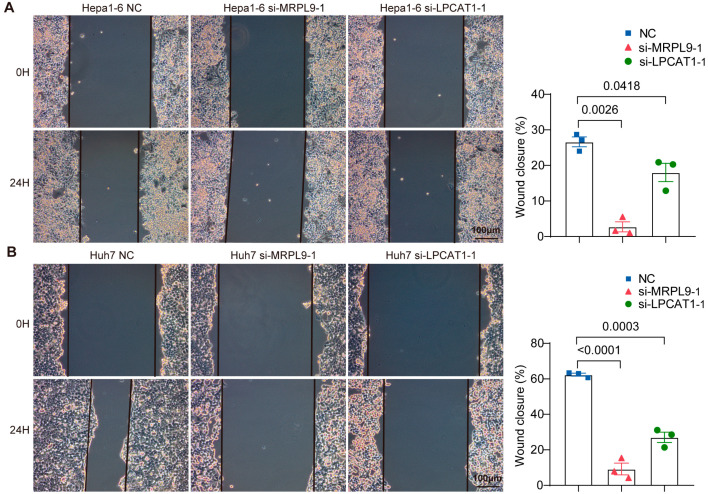
Effects of LPCAT1 and MRPL9 on hepatocellular carcinoma cell migration and apoptosis. (**A**) Representative phase-contrast images of scratch wounds at 0 and 24 h, along with quantitative analysis of wound closure in Hepa1-6 NC, siMRPL9-1, and siLPCAT1-1 cells. (**B**) Corresponding wound-healing images and quantification in Huh7 NC, siMRPL9-1, and siLPCAT1-1 cells. Black lines indicate wound edges. Scale bar: 100 μm. Mean ± SEM, *n* = 3/group.

We investigated the functional roles of LPCAT1 and MRPL9 in hepatocellular carcinoma cells. Hepa1-6 (mouse) and Huh7 (human) were employed to validate the biological effects of LPCAT1 and MRPL9 across species. Effective knockdown of LPCAT1 and MRPL9 in Hepa1-6 and Huh7 cells was confirmed by both Western blotting and qRT-PCR analyses (Figure 6A,B). Both immunofluorescence staining and flow cytometry showed significantly reduced EdU-positive cells in Hepa1-6 cells transfected with siMRPL9-1 or siLPCAT1-1, indicating that depletion of MRPL9 or LPCAT1 suppresses cell proliferation (Figure 6C,D). Consistent results were observed in Huh7 cells (Figure 6E,F). In addition, knockdown of MRPL9 or LPCAT1 significantly decreased cell migration rates (Figure 7A,B). These results suggest that silencing MRPL9 or LPCAT1 can inhibit HCC cell proliferation and migration.

### 3.7. Knockdown of LPCAT1 and MRPL9 Prevents HCC Progression Through AKT Phosphorylation

**Figure 8:**
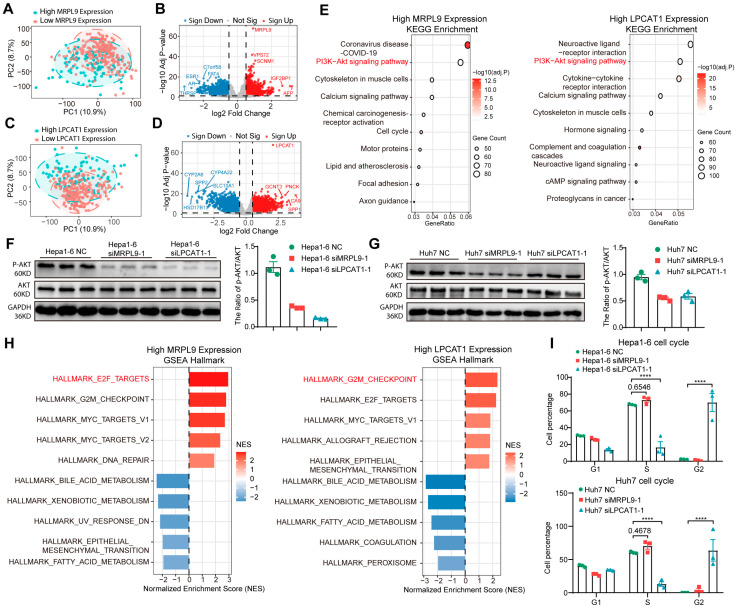
LPCAT1 and MRPL9 regulate hepatocellular carcinoma progression through the PI3K/AKT signaling pathway. (**A**) Principal component analysis (PCA) of MRPL9 high- and low-expression groups in the TCGA cohort. (**B**) Top 10 differentially expressed genes (DEGs) from the pairwise comparison of the MRPL9-high group versus the MRPL9-low group. Genes with |fold change| > 1 and adjusted *p* < 0.05 are highlighted in red (upregulated) and blue (downregulated). (**C**) PCA of LPCAT1 high- and low-expression groups in the TCGA cohort. (**D**) Top 10 DEGs from the pairwise comparison of the LPCAT1-high group versus the LPCAT1-low group. (**E**) Identification of the top 10 KEGG pathways most significantly upregulated in MRPL9/LPCAT1-high groups. (**F**) Western blot analysis of p-AKT, total AKT, and the p-AKT/AKT ratio in Hepa1-6 NC, Hepa1-6 siMRPL9-1, and Hepa1-6 siLPCAT1-1 cells. (**G**) Western blot analysis of p-AKT, total AKT, and the p-AKT/AKT ratio in Huh7 NC, Huh7 siMRPL9-1, and Huh7 siLPCAT1-1 cells. (**H**) Identification of the top 10 significantly upregulated (red) and downregulated (blue) Hallmark pathways from the MSigDB database in MRPL9/LPCAT1-high groups. Enrichment analysis was performed using gene set enrichment analysis (GSEA). (**I**) Flow cytometric analysis of cell cycle distribution in Hepa1-6 and Huh7 cells. Mean ± SEM, *n* = 3/group. **** *p* < 0.0001. Original western blots are presented in File S1.

In the TCGA cohort, HCC patients were divided into high- and low-expression groups based on the median levels of LPCAT1 and MRPL9. Principal component analysis (PCA) revealed distinct clustering between high- and low-expression groups for both MRPL9 and LPCAT1 (Figure 8A,C). DEGs were identified between the MRPL9 high- and low-expression groups (Figure 8B), and between the LPCAT1 high- and low-expression groups (Figure 8D). KEGG pathway enrichment analysis of significantly upregulated genes (*p* < 0.05 and fold change > 1) showed that the PI3K/AKT signaling pathway ranked among the top enriched pathways in both the MRPL9-high and LPCAT1-high groups (Figure 8E). Western blot analysis further confirmed that phosphorylation of AKT (p-AKT) was markedly reduced in MRPL9- and LPCAT1-knockdown Hepa1-6 and Huh7 cells compared with controls (Figure 8F,G), suggesting that LPCAT1 and MRPL9 may regulate HCC progression through the AKT phosphorylation. Pre-ranked gene set enrichment analysis (GSEA) using the Hallmark database identified the top 10 significantly enriched biological processes. Hallmark E2F targets (G1S) were significantly enriched in the MRPL9-high group, whereas Hallmark G2M checkpoint was significantly enriched in the LPCAT1-high group (Figure 8H). Consistent with these findings, MRPL9-knockdown cells showed a trend toward S-phase arrest (not statistically significant), while LPCAT1-knockdown cells exhibited a significant G2-phase arrest (Figure 8I). These findings suggest that LPCAT1 and MRPL9 regulate distinct cell-cycle checkpoints, thereby contributing to HCC progression.

## 4. Discussion

HCC remains a major clinical challenge owing to its complex pathophysiology and high mortality rates [1,2]. Accurate diagnosis and prognosis are crucial for guiding clinical decision-making and improving patient outcomes. Current diagnostic and prognostic approaches largely rely on imaging-based scoring systems, tumor staging, and a combination of clinical and laboratory indicators [10]. However, there is an urgent need for simple, accessible, and reliable biomarkers that can facilitate early prediction and improve risk stratification in HCC [27].

LPCAT1 and MRPL9 demonstrated robust prognostic performance in predicting HCC progression, with notably higher expression levels in HCC patients, particularly among non-survivors. Previous studies have also reported elevated LPCAT1 expression in HCC, supporting its role as an independent prognostic marker [28,29]. Additionally, recent research has shown that MRPL9 contributes to tumorigenesis and is associated with poor survival outcomes [30]. Moreover, MRPL9 demonstrated reliable diagnostic performance for distinguishing HCC patients. Taken together, these findings suggest that LPCAT1 and MRPL9 are not only potent prognostic biomarkers for HCC but also promising candidates for diagnostic and therapeutic applications, particularly MRPL9 for its superior diagnostic accuracy.

Developing a risk prognostic score model with high sensitivity and specificity is essential for improving patient outcomes in HCC. Previous studies established a mitochondria-related prognostic model based on six genes, which demonstrated favorable prognostic value for HCC at 1-, 2-, and 3-year in two cohorts [31]. In this study, we developed a simplified prognostic risk score model based on two significantly prognostic MRGs (LPCAT1 and MRPL9). We found that it exhibited robust prognostic accuracy for 1-year, 2-year, 3-year, 4-year, and 5-year survival in both the internal validation cohort and three independent external cohorts. Furthermore, it effectively distinguished patients into high- and low-risk groups, with these groups showing significantly different survival outcomes. Given the high recurrence rates and substantial tumor heterogeneity in HCC, current prognostic models and therapeutic strategies benefit only a limited subset of patients. Therefore, there is an urgent need to develop more precise and personalized prognostic tools to guide treatment decisions and improve patient outcomes [10,11,32,33]. However, further validation in larger, prospective, multicenter cohorts is necessary to confirm its clinical applicability and generalizability across diverse patient populations.

**Figure 9:**
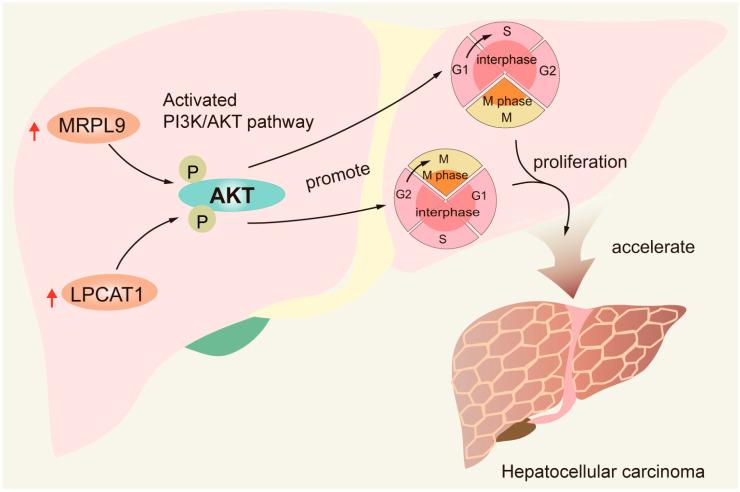
Schematic illustration of the proposed mechanism by which LPCAT1 and MRPL9 promote hepatocellular carcinoma progression. Elevated expression of LPCAT1 and MRPL9 enhances AKT phosphorylation. Activated AKT facilitates cell-cycle progression and tumor cell proliferation. Knockdown of MRPL9 or LPCAT1 reduces AKT phosphorylation, thereby arresting cells at the G1/S and G2/M phases, respectively.

Understanding the functional roles of LPCAT1 and MRPL9 in HCC progression is crucial for developing novel therapeutic strategies. Loss and dysfunction of hepatocyte-specific functions are recognized as maladaptive processes that drive the progression of liver diseases [34]. In this study, pathways related to metabolic dysfunction were significantly enriched in the high-risk group defined by the 2-MRG risk score model, which is consistent with the poorer prognosis observed in these patients. Functional enrichment analysis of LPCAT1 or MRPL9 high- and low-expression groups in the TCGA cohort revealed significant activation of the PI3K/AKT signaling pathway in the high-expression groups, suggesting that these genes may participate in the regulation of PI3K/AKT signaling. Consistently, in vitro experiments demonstrated that knockdown of LPCAT1 or MRPL9 inhibited HCC cell proliferation and migration and significantly reduced the protein expression level of phosphorylated AKT (p-AKT). The PI3K/AKT signaling pathway is widely recognized as a critical oncogenic pathway that promotes tumor cell proliferation and metastatic progression. AKT is a serine/threonine kinase that functions as a central downstream effector of PI3K, regulating multiple cellular processes including cell proliferation, survival, metabolism, and migration. Upon activation by phosphorylation, AKT promotes tumor progression by inhibiting apoptosis, enhancing cell-cycle progression, and promoting metabolic reprogramming [35,36]. In this study, we also found that knockdown of MRPL9 or LPCAT1 induced G1/S arrest or G2/M arrest, respectively, further supporting their involvement in AKT-mediated regulation of cell-cycle progression in HCC. These findings suggest that LPCAT1 and MRPL9 promote HCC progression, at least in part, by enhancing AKT phosphorylation and modulating cell-cycle checkpoints, underscoring their potential as therapeutic targets (Figure 9).

The sensitivity of the 2-MRG risk score model to systemic therapy is crucial for improving clinical outcomes and advancing personalized treatment strategies for HCC patients. In recent years, novel targeted therapies and immune checkpoint inhibitors have been approved as systemic treatments for patients with unresectable HCC or those ineligible for locoregional therapies [9,10]. Previous studies have demonstrated that elevated TMB leads to the generation of more neoantigens, which are processed and presented by major histocompatibility complex (MHC) molecules to T cells, thereby eliciting an anti-tumor immune response [37]. In our study, the TMB in the high-risk group was significantly higher than that in the low-risk group. This suggests that patients in the high-risk group may exhibit heightened immune responsiveness and could potentially derive greater benefit from immunotherapy. Furthermore, TP53 was the most frequently mutated gene in the high-risk group, whereas CTNNB1 mutations were predominant in the low-risk group. Prior research has shown that TP53 mutations are associated with poor prognosis and unfavorable survival outcomes in HCC, whereas CTNNB1-mutated tumors are often well-differentiated and linked to more favorable clinical features [38,39], further supporting the observation of a better prognosis in the low-risk group. Additionally, the expression levels of inflammation-related genes were significantly elevated in the high-risk group. Several immune checkpoint-related genes, including CTLA4 and CD47, were significantly upregulated in the high-risk group, indicating a potentially active immune regulatory environment. These immune checkpoint expression patterns indicate that the high-risk group may harbor a more immunogenic tumor microenvironment, potentially making these patients more responsive to immune checkpoint blockade therapy. Consequently, it is believed that HCC patients classified as high risk based on the 2-MRG model may derive greater therapeutic benefit from immunotherapy, highlighting the model’s utility in guiding treatment decisions and advancing precision oncology in HCC.

## 5. Conclusions

In summary, we developed a novel prognostic model for HCC that is based on two key mitochondrial genes, LPCAT1 and MRPL9. This simple risk score has demonstrated strong prognostic accuracy and may help guide immunotherapy selection. An elevated risk score may reflect underlying metabolic dysfunction in HCC. Moreover, knockdown of LPCAT1 or MRPL9 has been shown to suppress HCC progression by regulating the AKT phosphorylation and cell-cycle checkpoints, underscoring their potential as therapeutic targets.

## Data Availability

All data used to generate these results are available in the main text and Supporting Information.

## Supplementary Materials

The following supporting information can be downloaded at https://www.mdpi.com/article/10.3390/cancers18071144/s1. Figure S1. LASSO regression analysis identified ten featured mitochondrial-related genes associated with patient survival; Figure S2. Volcano plot depicting the significant differentially expressed genes (DEGs) from the pairwise comparison of the high-risk group versus the low-risk group.; Table S1: Prognostic genes in Univariate Cox regression analysis of HCC transcriptomic data from the TCGA cohort; Table S2: Prognostic genes in Univariate Cox regression analysis of HCC transcriptomic data from the ICGC- LIHC cohort; Table S3: Primers used in this study; Table S4: Antibodies used for Western blot; File S1: Original western blots.

## Abbreviations

The following abbreviations are used in this manuscript:

HCC	Hepatocellular carcinoma
MRGs	Mitochondrial-related genes
LPCAT1	Lysophosphatidylcholine Acyltransferase 1
MRPL9	Mitochondrial Ribosomal Protein L9
BCLC	Barcelona Clinic Liver Cancer
TCGA	The Cancer Genome Atlas
LIHC	Liver hepatocellular carcinoma
ICGC	International Cancer Genome Consortium
GEO	Gene Expression Omnibus
OS	overall survival
ROC	Receiver operating characteristic
DEGs	Differentially expressed genes
