# Supplementary material for: LPCAT1 and MRPL9 Promote Hepatocellular Carcinoma Progression via AKT Phosphorylation and Define a Mitochondrial Prognostic Model
Source: Cancers (Basel). 2026 Apr 2;18(7):1144. doi: 10.3390/cancers18071144 (PMC13072332; doi:10.3390/cancers18071144)
Supplement: Supplementary file 1 [file cancers-18-01144-s001.zip › SUPPLEMENTARY MATERIALS.pdf]

LPCAT1 and MRPL9 suppress hepatocellular carcinoma progression via PI3K/AKT signaling and develop a mitochondrial prognostic model

SUPPLEMENTARY FIGURES

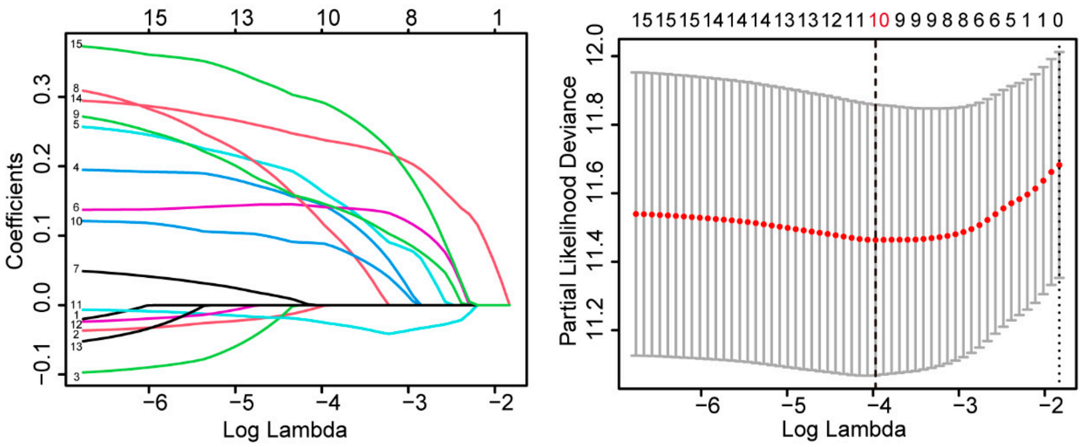

Figure S1. LASSO regression analysis identified ten featured mitochondrial-related genes associated with patient survival.

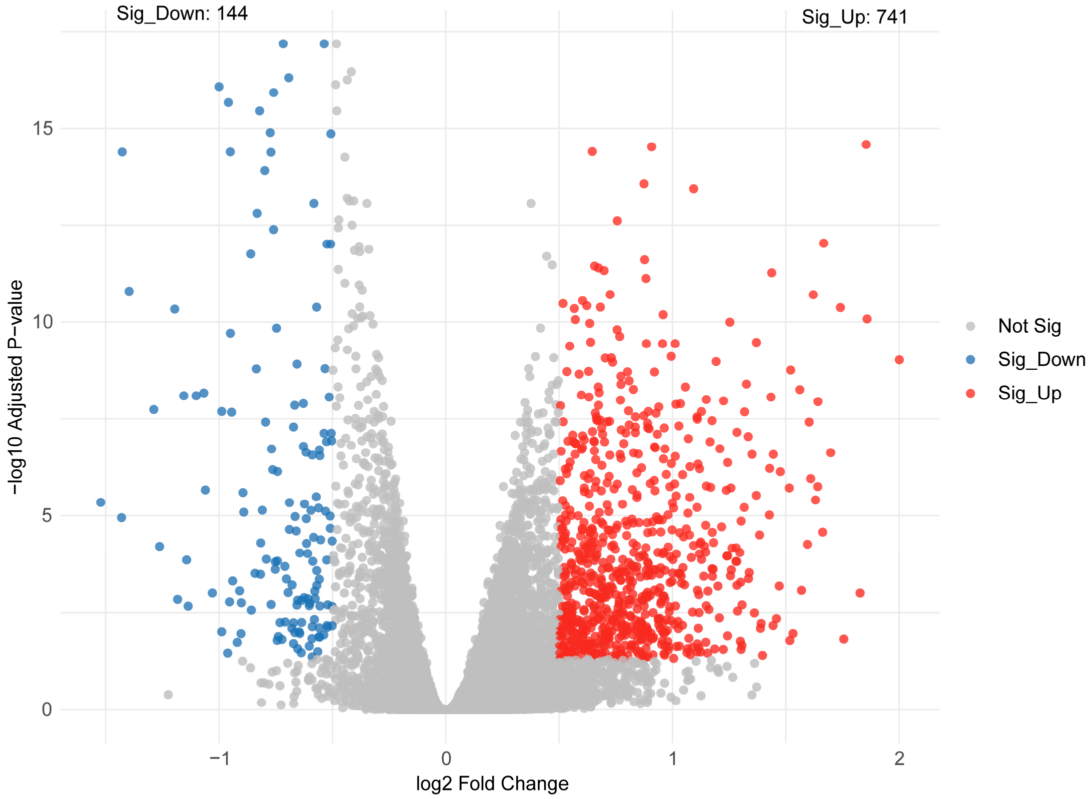

Figure S2. Volcano plot depicting the significant differentially expressed genes (DEGs) from the pairwise comparison of the high-risk group versus the low-risk group. Genes with significant differential expression (|log2-fold change| > 0.5; adjusted p-value < 0.05) are highlighted in red (upregulated) and blue (downregulated).
