# Supplementary material for: LPCAT1 and MRPL9 Promote Hepatocellular Carcinoma Progression via AKT Phosphorylation and Define a Mitochondrial Prognostic Model
Source: Cancers (Basel). 2026 Apr 2;18(7):1144. doi: 10.3390/cancers18071144 (PMC13072332; doi:10.3390/cancers18071144)
Supplement: Supplementary file 1 [file cancers-18-01144-s001.zip › Table S3.pdf]

Table S3: Primers used in this study

**Symbol**		**Sequence gene**	**ID**
hMRPL9	Forward	5’-GTTACCAGAAGAGCCTATCACAC-3’	65005
	Reverse	5’-CTCTCACAGTATCAAGCCCATTT-3’	
hLPCAT1	Forward	5’-ACATCCCGATCTGGGGAACT-3’	79888
	Reverse	5’-GGCCACTTTCCGTTGGACT-3’	
hGAPDH	Forward	5’-CTCTCTGCTCCTCCTGTTCG-3’	2597
	Reverse	5’-ACGACCAAATCCGTTGACTC-3’	
mMRPL9	Forward	5’-AGGCATCCGGGAGCTATTC-3’	78523
	Reverse	5’-TTGTCTTTGGGCCGGTGTTT-3’	
mLPCAT1	Forward	5’-GGCTCCTGTTCGCTGCTTT-3’	210992
	Reverse	5’-TTCACAGCTACACGGTGGAAG-3’	
mGAPDH	Forward	5’-CATCACTGCCACCCAGAAGACTG-3’	14433
	Reverse	5’-ATGCCAGTGAGCTTCCCGTTCAG-3’	
hsiRNA-MRPL9-1		5’-GGUAAAUGGGCUUGAUACUTT-3’<br>5’-AGUAUCAAGCCCAUUUACCTT-3’	
hsiRNA-MRPL9-2		5’-GCUGAACCCUGAAAUAGUUTT-3’<br>5’-AACUAUUUCAGGGUUCAGCTT-3’	
hsiRNA-LPCAT1-1		5’-CCAGAAGGAACUUGUACAATT-3’<br>5’-UUGUACAAGUUCCUUCUGGTT-3’	

hsiRNA-LPCAT1-2	5’-GUGUCUCCGUGACUGACUATT-3’<br>5’-UAGUCAGUCACGGAGACACTT-3’
msiRNA-MRPL9-1	5’-CCGCGAAUUGAAGGGAGUATT-3’<br>5’-UACUCCCUUCAAUUCGCGGTT-3’
msiRNA-MRPL9-2	5’-GAGAAGCUGUCAUCUAGAGTT-3’<br>5’-CUCUAGAUGACAGCUUCUCTT-3’
msiRNA-LPCAT1-1	5’-GGGAGAAAGAAUUCUUGUATT-3’<br>5’-UACAAGAAUUCUUUCUCCCTT-3’
msiRNA-LPCAT1-2	5’-CUGGGUGUAUCAGAAUUAATT-3’<br>5’-UUAAUUCUGAUACACCCAGTT-3’
