# Supplementary material for: LPCAT1 and MRPL9 Promote Hepatocellular Carcinoma Progression via AKT Phosphorylation and Define a Mitochondrial Prognostic Model
Source: Cancers (Basel). 2026 Apr 2;18(7):1144. doi: 10.3390/cancers18071144 (PMC13072332; doi:10.3390/cancers18071144)
Supplement: Supplementary file 1 [file cancers-18-01144-s001.zip › Table S4.pdf]

Table S4: Antibodies used for Western blot

**Antibodies**	**Company/Cat Numb**	**Dilution**
**Primary antibodies**		
MRPL9	ABclonal/ A25241	1:1000
LPCAT1	ABclonal/ A4987	1:1000
p-AKT	ABclonal/ AP1208	1:1000
AKT	ABclonal/ A18675	1:1000
GAPDH	HUABIO/ ET1601-4	1:100000
**Secondary antibodies**		
Goat anti-rabbit IgG (HRP)	ABclonal/ AS014	1:50000
Goat anti-mouse IgG (HRP)	ABclonal/ AS003	1:50000
