# Supplementary figures and images for: LPCAT1 and MRPL9 Promote Hepatocellular Carcinoma Progression via AKT Phosphorylation and Define a Mitochondrial Prognostic Model
Source: Cancers (Basel). 2026 Apr 2;18(7):1144. doi: 10.3390/cancers18071144 (PMC13072332; doi:10.3390/cancers18071144)

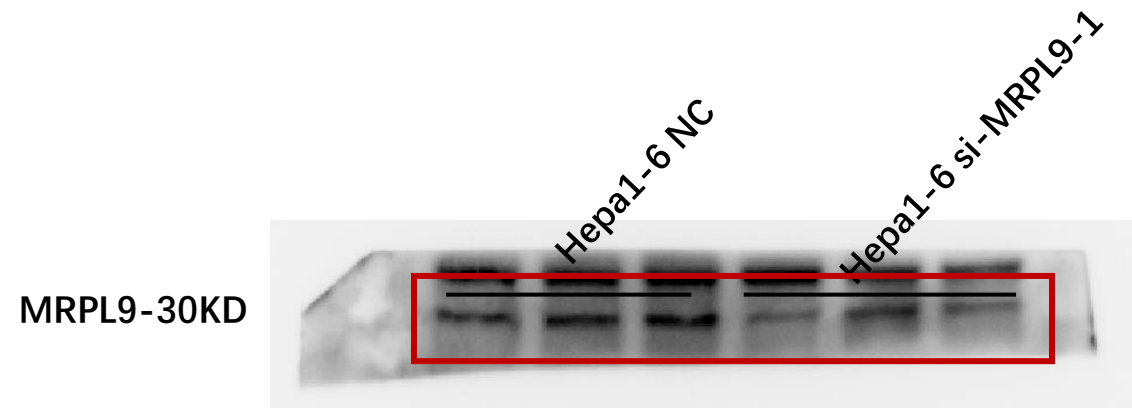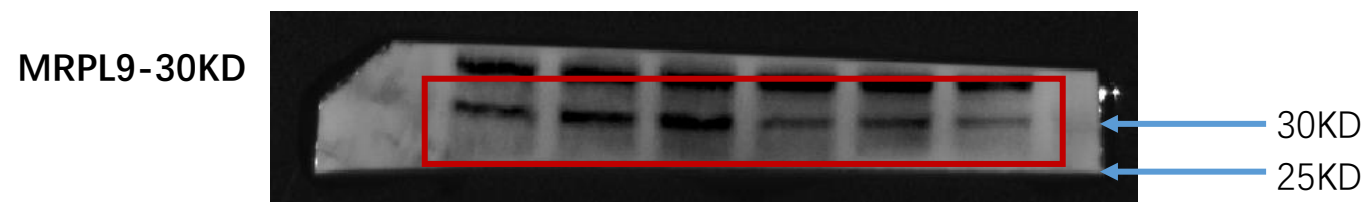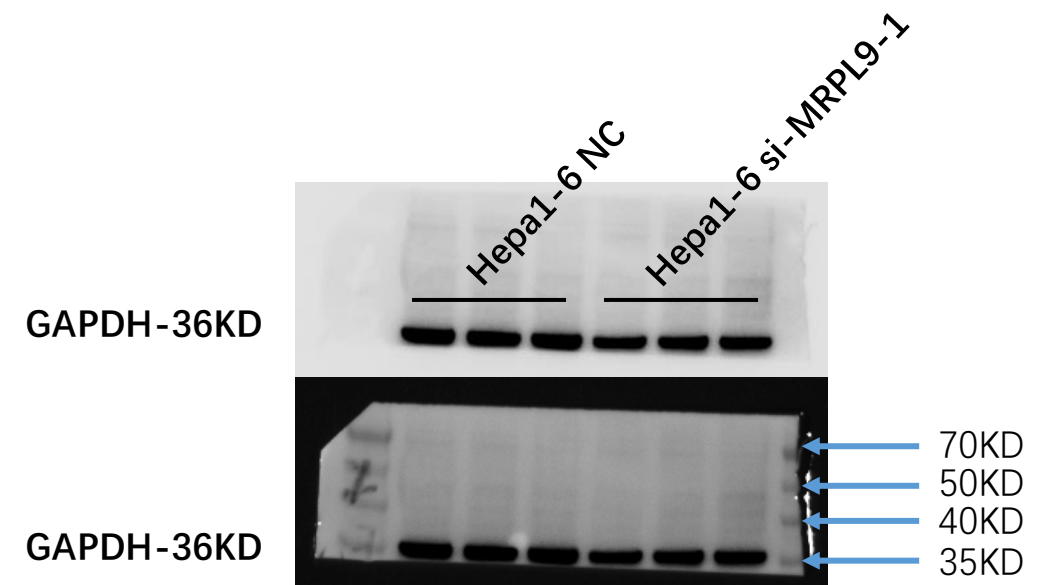

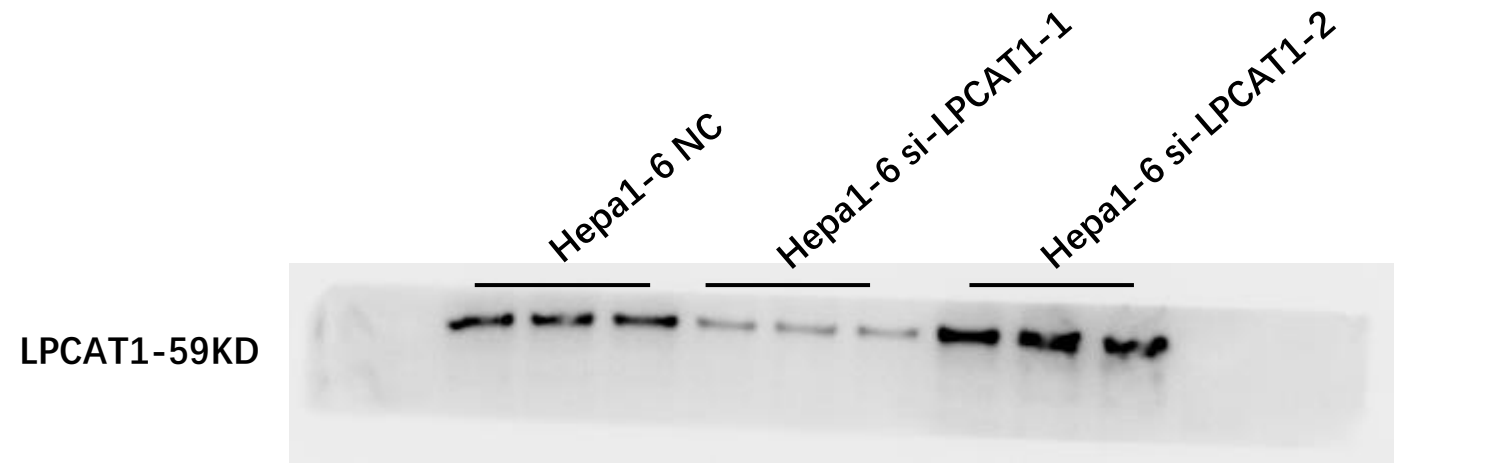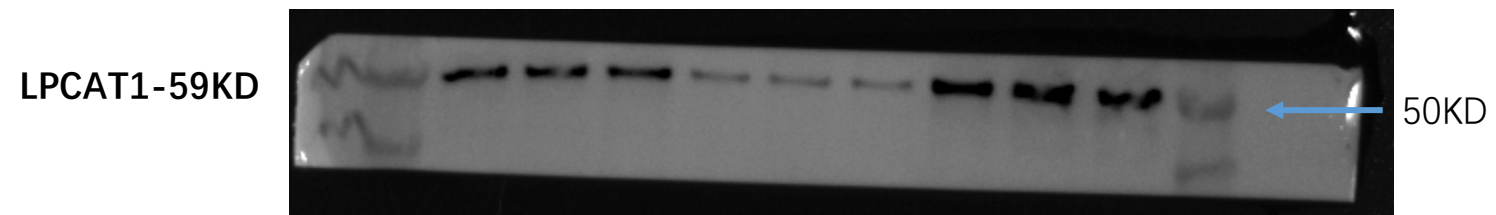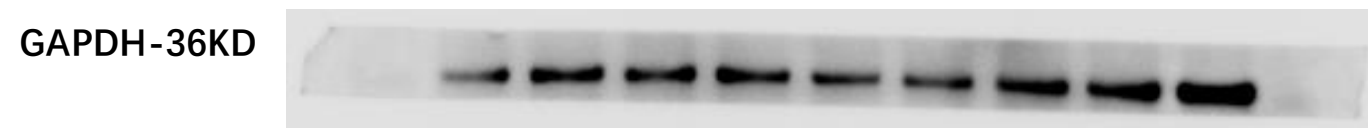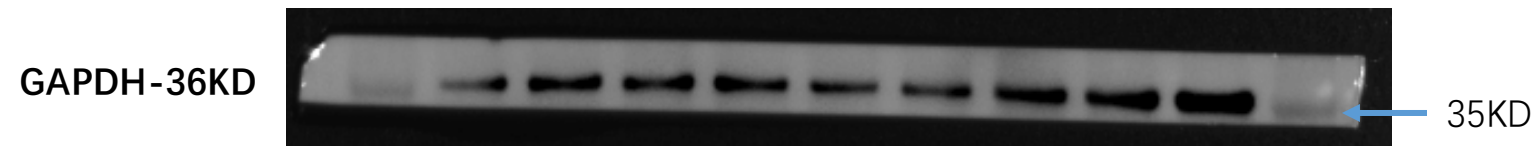

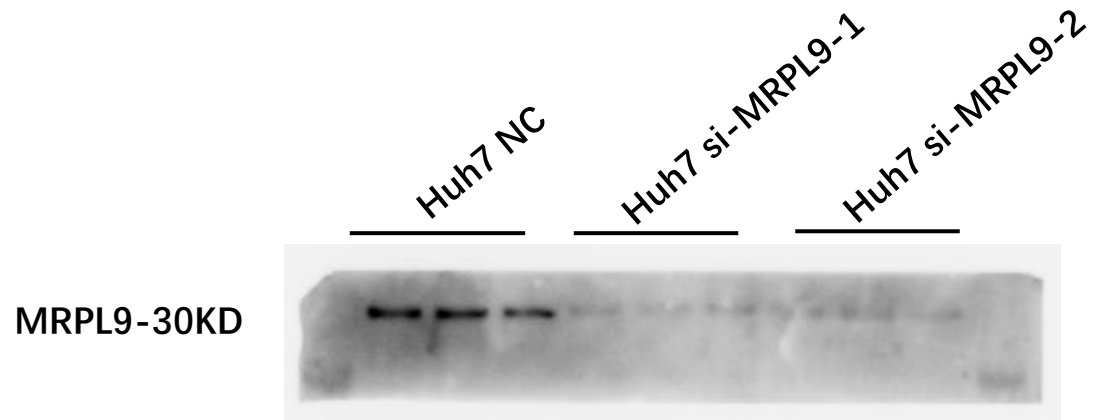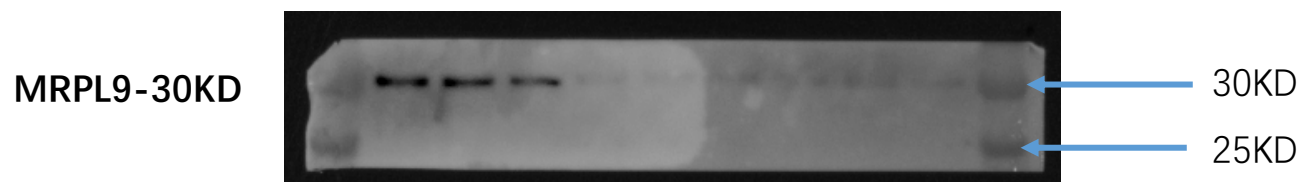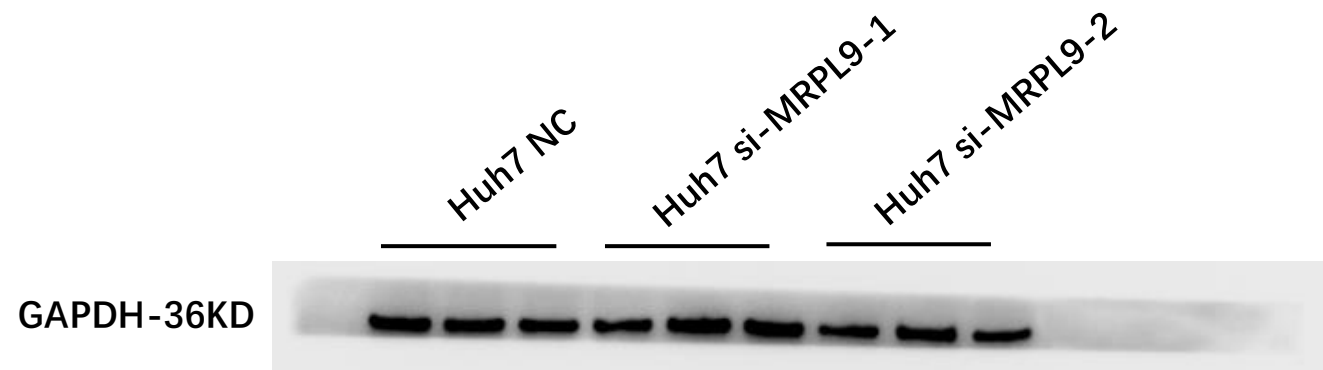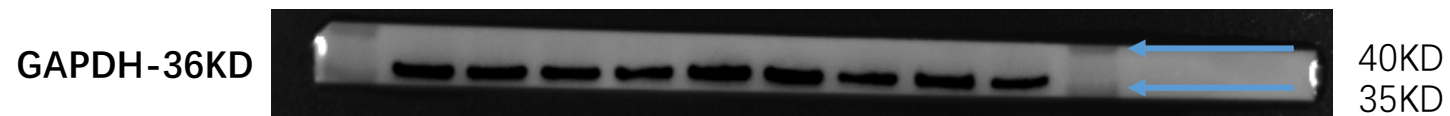

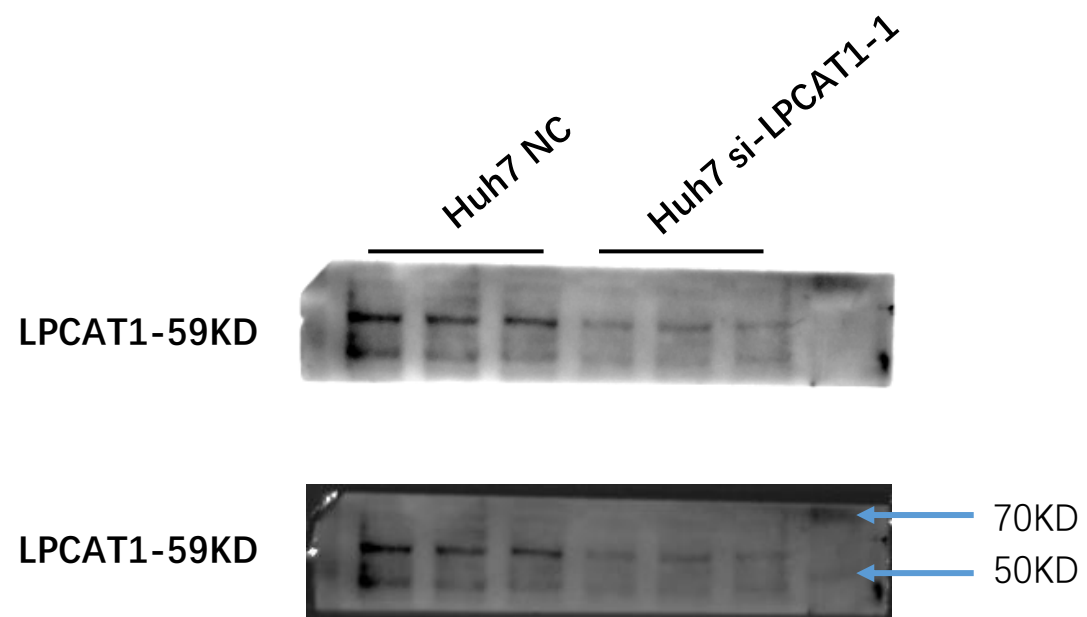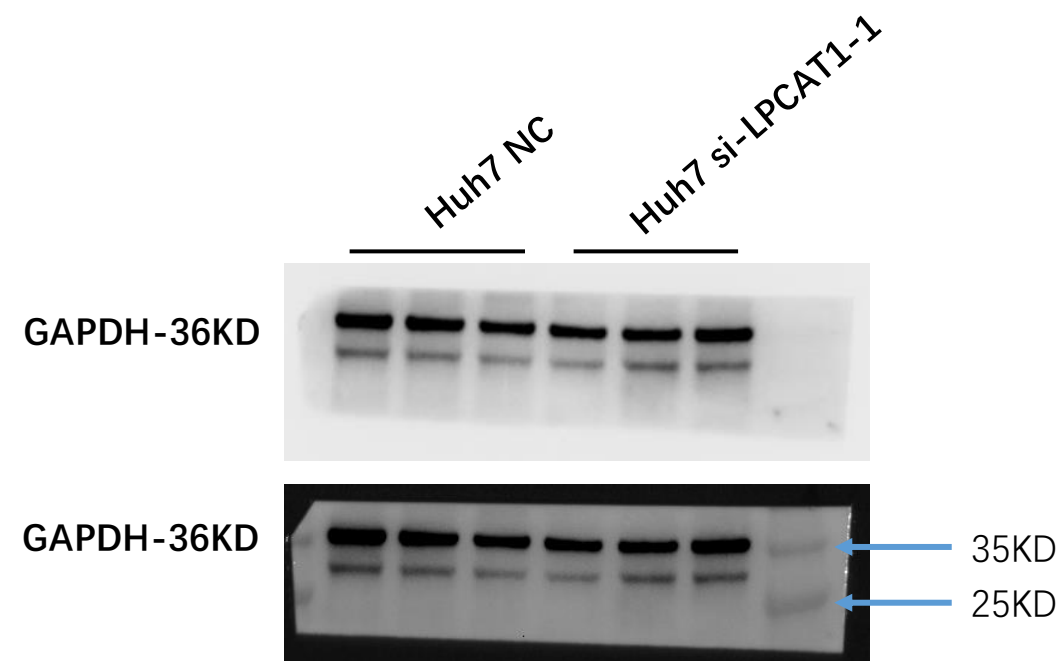

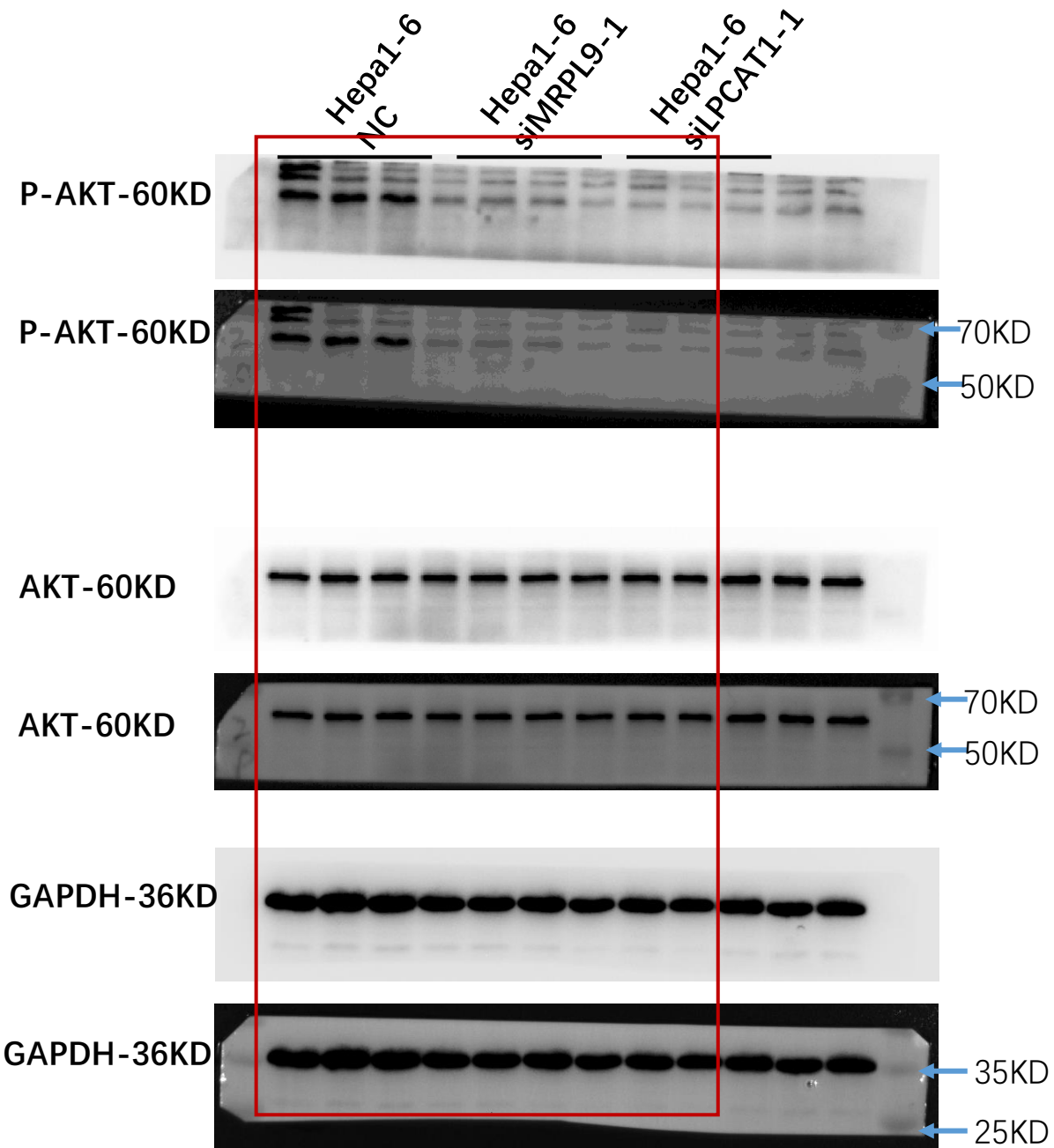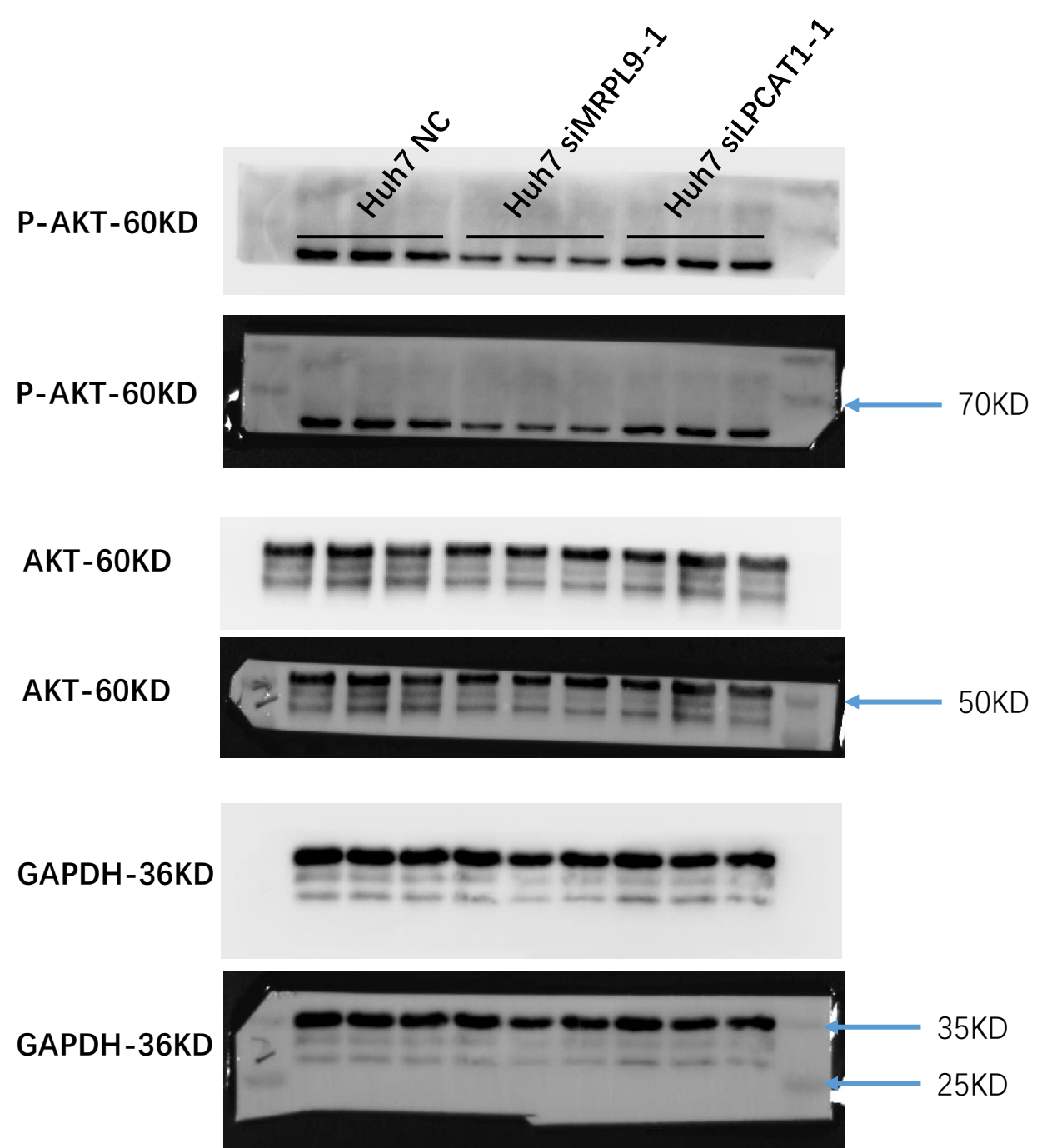

Supplement: Supplementary file 1 [file cancers-18-01144-s001.zip › File S1.pdf]
